# Progress Along the Continuum of HIV Care Among Blacks with Diagnosed HIV— United States, 2010

**Published:** 2014-02-07

**Authors:** Y. Omar Whiteside, Stacy M. Cohen, Heather Bradley, Jacek Skarbinski, H. Irene Hall, Amy Lansky

**Affiliations:** 1 Division of HIV/AIDS Prevention, National Center for HIV/AIDS, Viral Hepatitis, STD, and TB Prevention, CDC

The goals of the National HIV/AIDS Strategy are to reduce new human immunodeficiency virus (HIV) infections, increase access to care and improve health outcomes for persons living with HIV, and reduce HIV-related health disparities (1). Recently, by executive order, the HIV Care Continuum Initiative was established, focusing on accelerating federal efforts to increase HIV testing, care, and treatment (2). Blacks are the racial group most affected, comprising 44% of new infections (3) and also 44% of all persons living with HIV infection (4). To achieve the goals of NHAS, and to be consistent with the HIV Care Continuum Initiative, blacks with HIV need high levels of care and viral suppression (5–7). Achieving these goals calls for 85% of blacks with diagnosed HIV to be linked to care, 80% to be retained in care, and the proportion with an undetectable viral load (VL) to increase 20% by 2015 (1). Analysis of data from the National HIV Surveillance System (NHSS)* and the Medical Monitoring Project (MMP)† regarding progress along the HIV care continuum during 2010 for blacks with diagnosed HIV infection indicated that 74.9% of HIV-diagnosed blacks were linked to care, 48.0% were retained in care, 46.2% were prescribed antiretroviral therapy (ART), and 35.2% had achieved viral suppression. Black males had lower levels of care and viral suppression than black females at each step along the HIV care continuum; in addition, levels of care and viral suppression for blacks aged <25 years were lower than those for blacks aged ≥25 years at each step of the continuum. These data demonstrate the need for implementation of interventions and public health strategies that increase linkage to care and consistent ART among blacks, particularly black males and black youths.

Data from NHSS in 2010 reported to CDC through December 2012 were used to determine the numbers of blacks aged ≥13 years newly diagnosed with HIV and living with diagnosed HIV and the numbers and percentages linked to care and retained in care. Nineteen jurisdictions met the criteria for the collection and reporting of CD4+ T-lymphocyte (CD4) and VL test results,§ which are the data needed to assess linkage and retention in care. Linkage to care¶ was calculated among blacks with new HIV diagnoses during 2010 who resided in any of the 19 jurisdictions at diagnosis. Retention in care** was assessed among blacks with HIV diagnosed by December 31, 2009, who resided in any of the 19 jurisdictions at the time of diagnosis, and were alive on December 31, 2010, (i.e., persons living with diagnosed HIV). Data were statistically adjusted for missing HIV transmission categories (8).

Data from MMP were used to estimate ART prescription†† and viral suppression§§ among blacks aged ≥18 years using methods that have been described previously (5). The MMP values are weighted national estimates of the numbers of blacks who received medical care during January–April 2010 and had documentation of ART prescription and viral suppression. Percentages were calculated among blacks whose HIV infection was diagnosed by December 31, 2009, and who were alive on December 31, 2010, in the United States and Puerto Rico (denominators were based on NHSS data). Data analyses were limited to 2010, the most recent year data were available for persons living with HIV infection.

Of the 8,261 blacks with HIV infection diagnosed during 2010 in the 19 jurisdictions, 6,186 (74.9%) were linked to care ≤3 months after HIV diagnosis (Table 1). Among males, 72.3% were linked to care, compared with 81.3% of females. Persons aged 13–24 years had the highest number of diagnoses of any age group, but the lowest percentage of linkage to care (68.8%); linkage increased with age group. By transmission category, males with infection attributed to male-to-male sexual contact had the lowest percentage of linkage to care (71.6%); the highest percentage was among females with infection attributed to injection drug use (82.4%), followed by females with infection attributed to heterosexual contact (81.1%).

Among the 153,581 blacks aged ≥13 years living with diagnosed HIV on December 31, 2010, in 19 jurisdictions, 48.0% were retained in care (Table 2). Of these, a lower percentage of males (46.5%) than females (50.9%) were retained in care. By age group, persons aged 25–34 years had the lowest percentage retained in care (42.8%), followed by persons aged 13–24 years (45.1%). By transmission category, the lowest percentage retained in care was among males with infection attributed to injection drug use (43.9%); the highest percentages were among females with infection attributed to injection drug use (50.9%) and females with infection attributed to heterosexual contact (50.6%).

Of 353,653 blacks aged ≥18 years living with diagnosed HIV on December 31, 2010, in the United States and Puerto Rico, 163,515 (46.2%) had an ART prescription (Table 3). Of these, a higher percentage of females (50.8%) than males (43.7%) had ART prescribed. Prevalence of ART prescription increased with age group; prevalence was 20.8% among blacks aged 18–24 years and 57.4% among those aged ≥55 years. The lowest level of ART prescription by transmission category was among males with infection attributed to injection drug use (34.0%); the highest level was among females with infection attributed to heterosexual contact (51.4%).

Of blacks living with diagnosed HIV in the United States and Puerto Rico, 35.2% achieved viral suppression at their most recent test. Of these persons, a higher percentage of females had suppressed VL (39.8%) than males (32.7%). Persons aged 18–24 years had the lowest level of viral suppression (18.3%) among all age groups. By transmission category, males with infection attributed to injection drug use had the lowest level of viral suppression (22.2%), and females with infection attributed to heterosexual contact had the highest level (41.3%).

## Editorial Note

The results of the analysis described in this report indicate that, in 2010, among blacks with HIV diagnoses of all age groups and both sexes, 74.9% were linked to care, 48.0% were retained in care, 46.2% were prescribed ART, and 35.2% had achieved viral suppression. Improving health outcomes for blacks living with HIV infection is necessary to reduce HIV infection in the United States.

Blacks with HIV might not seek, receive, or adhere to HIV care or achieve viral suppression for reasons including lack of health insurance, poverty, and stigma (9). HIV programs that focus on care and treatment for blacks might strengthen efforts to link and retain HIV-infected persons in care and promote adherence to medication to achieve optimal health outcomes. Evidence-based interventions with demonstrated efficacy in scientific studies and effectiveness in practice settings also might be considered (10).

Among black persons with HIV in the United States, males had a lower prevalence than females of linkage to care, retention in care, ART prescription, and viral suppression. The youngest age group among blacks had lower percentages than other age groups of linkage to care, ART prescription, and viral suppression. In addition to interventions to ensure that all persons with HIV receive optimal care to improve health outcomes, targeted strategies for groups such as black males and black youths might be needed to achieve improvements at each step of the continuum.

The findings in this report are subject to at least two limitations. First, analyses based on NHSS data are limited to 19 jurisdictions with complete reporting of all levels of CD4 and VL test results; data from these areas represent approximately 44% of all blacks living with diagnosed HIV on December 31, 2010, in the United States and might not be representative of all blacks in the United States. Second, certain analyses in this study are based on different populations, and the results cannot be compared because linkage to care and retention in care were based on data for persons aged ≥13 years from 19 jurisdictions, whereas ART prescription and viral suppression were based on weighted estimates of persons receiving care aged ≥18 years from the United States and Puerto Rico.

What is already known on this topic?

Blacks account for 44% of persons living with human immunodeficiency virus (HIV) but only 12% of the population in the United States. The percentages of blacks linked to care, retained in care, taking antiretroviral medications, and achieving viral suppression have been lower than other racial/ethnic groups.

What is added by this report?

This is the first known report to describe the continuum of HIV care among blacks in the United States. The results of this analysis of 2010 data indicate that 74.9% of HIV-infected blacks were linked to care, 48.0% were retained in care, 46.2% were prescribed antiretroviral therapy, and 35.2% had achieved viral suppression. Black males had lower levels of care and viral suppression than black females at each step along the HIV care continuum, and levels of care and viral suppression for blacks aged <25 years were lower than those for blacks aged ≥25 years.

What are the implications for public health practice?

Increasing the proportion of black persons living with HIV who are receiving care is critical for achieving the goals of the National HIV/AIDS Strategy to reduce new infections, improve health outcomes, and decrease health disparities. Among blacks, targeted strategies for different groups, such as males and youths, might be needed to achieve improvements at each step of the HIV care continuum.

CDC and its partners are pursuing a high-impact prevention¶¶ approach to advance the goals of the National HIV/AIDS Strategy and maximize the effectiveness of current HIV prevention and care methods. Testing is a critical first step of entry into the HIV continuum of care. CDC supports HIV testing projects that focus on blacks. CDC also supports multiple projects to optimize outcomes along the continuum of care, such as the Care and Prevention in the United States*** demonstration project, which seeks to increase linkage to, retention in, and return to care for all HIV-infected persons, including racial and ethnic minorities, with the goal of reducing HIV-related morbidity and mortality by addressing social, economic, clinical, and structural factors influencing HIV health outcomes. The results of the analyses described in this report underscore the need for enhanced linkage to care, retention in care, and viral suppression for blacks, particularly black males and black youths. Focusing prevention and care efforts on populations that bear a disproportionate burden of HIV disease could lead to reductions in HIV incidence and health inequities and help achieve the goals of the National HIV/AIDS Strategy.

## Figures and Tables

**TABLE 1:** Linkage to HIV medical care within 3 months after HIV diagnosis during 2010,* among blacks aged ≥13 years, by selected characteristics — National HIV Surveillance System, 19 jurisdictions,† United States

Characteristic	No. HIV diagnoses	Linkage to care§	

		No.	(%)
**Sex**			
Male	5,927	4,288	(72.3)
Female	2,334	1,898	(81.3)
**Age group at diagnosis (yrs)**			
13–24	2,238	1,539	(68.8)
25–34	2,147	1,569	(73.1)
35–44	1,648	1,287	(78.1)
45–54	1,511	1,213	(80.3)
≥55	717	578	(80.6)
**Transmission category** ¶			
Male-to-male sexual contact	4,348	3,115	(71.6)
Injection drug use			
Male	466	347	(74.6)
Female	323	266	(82.4)
Male-to-male sexual contact and injection drug use	168	124	(73.6)
Heterosexual contact**			
Male	939	696	(74.2)
Female	2,008	1,628	(81.1)
**Total** ††	**8,261**	**6,186**	**(74.9)**

**Abbreviation:** HIV = human immunodeficiency virus.

* Data include persons with a diagnosis of HIV infection regardless of stage of disease at diagnosis.

† The 19 jurisdictions were California (Los Angeles County and San Francisco only), Delaware, District of Columbia, Georgia, Hawaii, Illinois, Indiana, Iowa, Louisiana, Michigan, Minnesota, Missouri, Nebraska, New Hampshire, New York, North Dakota, South Carolina, West Virginia, and Wyoming.

§ One or more CD4+ T-lymphocyte or viral load test within 3 months after HIV diagnosis.

¶ Data statistically adjusted to account for missing transmission categories.

** Heterosexual contact with a person known to have, or to be at high risk for, HIV infection.

†† Includes 10 persons with diagnosed infection attributed to hemophilia, blood transfusion, perinatal exposure, and risk factor not reported or not identified.

**TABLE 2:** Retention in HIV medical care among blacks aged ≥13 years with HIV infection diagnosed by December 31, 2009,* who were alive on December 31, 2010, by selected characteristics — National HIV Surveillance System, 19 jurisdictions,† United States

		Retained in care in 2010§	
		
Characteristic	No.	No.	(%)
**Sex**			
Male	101,836	47,324	(46.5)
Female	51,745	26,332	(50.9)
**Age group on December 31, 2010 (yrs)**			
13–24	9,715	4,383	(45.1)
25–34	23,718	10,159	(42.8)
35–44	41,948	19,640	(46.8)
45–54	50,643	25,637	(50.6)
≥55	27,557	13,837	(50.2)
**Transmission category** ¶			
Male-to-male sexual contact	57,942	26,852	(46.3)
Injection drug use			
Male	19,637	8,619	(43.9)
Female	13,575	6,910	(50.9)
Male-to-male sexual contact and injection drug use	7,582	3,768	(49.7)
Heterosexual contact**			
Male	15,305	7,407	(48.4)
Female	36,666	18,563	(50.6)
Other††			
Male	1,371	677	(49.4)
Female	1,504	859	(57.1)
**Total**	**153,581**	**73,656**	**(48.0)**

**Abbreviation:** HIV = human immunodeficiency virus.

* Data include persons with a diagnosis of HIV infection regardless of stage of disease at diagnosis.

† The 19 jurisdictions were California (Los Angeles County and San Francisco only), Delaware, District of Columbia, Georgia, Hawaii, Illinois, Indiana, Iowa, Louisiana, Michigan, Minnesota, Missouri, Nebraska, New Hampshire, New York, North Dakota, South Carolina, West Virginia, and Wyoming.

§ Two or more CD4+ T-lymphocyte or viral load test performed at least 3 months apart during 2010.

¶ Data statistically adjusted to account for missing transmission categories.

** Heterosexual contact with a person known to have, or to be at high risk for, HIV infection.

†† Includes persons with diagnosed infection attributed to hemophilia, blood transfusion, perinatal exposure, or risk factor not reported or not identified.

**TABLE 3:** Antiretroviral prescription and viral suppression among blacks aged ≥18 years with HIV infection diagnosed by December 31, 2009,* who were alive on December 31, 2010, by selected characteristics — National HIV Surveillance System, Medical Monitoring Project, United States and Puerto Rico

		Antiretroviral therapy (ART) prescription§		Viral suppression¶	
			
Characteristic	No.†	No.	(%)	No.	(%)
**Sex**					
Male	228,794	100,013	(43.7)	74,753	(32.7)
Female	124,859	63,461	(50.8)	49,671	(39.8)
**Age group at interview (yrs)**					
18–24	19,994	4,161	(20.8)	3,666	(18.3)
25–34	56,711	20,890	(36.8)	14,395	(25.4)
35–44	100,232	42,220	(42.1)	32,525	(32.4)
45–54	117,235	62,077	(53.0)	46,866	(40.0)
≥55	59,481	34,167	(57.4)	27,011	(45.4)
**Transmission category** **					
Male-to-male sexual contact	123,819	58,276	(47.1)	45,813	(37.0)
Injection drug use					
Male	43,347	14,733	(34.0)	9,610	(22.2)
Female	28,703	14,289	(49.8)	10,407	(36.3)
Male-to-male sexual contact and injection drug use	16,346	8,065	(49.3)	6,096	(37.3)
Heterosexual contact††					
Male	43,392	18,287	(42.1)	12,854	(29.6)
Female	94,131	48,429	(51.4)	38,918	(41.3)
Other transmission§§	3,915	1,434	(36.6)	768	(19.6)
**Total** ¶¶	**353,653**	**163,515**	**(46.2)**	**124,465**	**(35.2)**

**Abbreviation:** HIV = human immunodeficiency virus.

* Data include persons with a diagnosis of HIV infection regardless of stage of disease at diagnosis.

† National HIV Surveillance System estimates for United States and Puerto Rico.

§ Medical Monitoring Project estimates for United States and Puerto Rico for persons who received medical care during January–April 2010 and who had documentation of ART prescription in the medical record.

¶ Medical Monitoring Project estimates for United States and Puerto Rico for persons who received medical care during January–April 2010 and whose most recent HIV viral load in the preceding 12 months was undetectable or ≤200 copies/mL.

** Data statistically adjusted to account for missing transmission categories.

†† Heterosexual contact with a person known to have, or to be at high risk for, HIV infection.

§§ Includes persons with diagnosed infection attributed to hemophilia, blood transfusion, perinatal exposure, or risk factor not reported or not identified.

¶¶ Estimates might not sum to total.

## Footnotes

* NHSS is the primary source for monitoring HIV trends in the United States. The system collects, analyzes, and disseminates information about new and existing cases of HIV infection.

† MMP is a supplemental HIV surveillance system designed to produce nationally representative estimates of the prevalence of behavioral and clinical characteristics among HIV-infected adults aged ≥18 years receiving medical care in the United States and Puerto Rico.

§ The 19 jurisdictions were California (Los Angeles County and San Francisco only), Delaware, District of Columbia, Georgia, Hawaii, Illinois, Indiana, Iowa, Louisiana, Michigan, Minnesota, Missouri, Nebraska, New Hampshire, New York, North Dakota, South Carolina, West Virginia, and Wyoming. The criteria for complete reporting were as follows: 1) the jurisdiction’s laws or regulations required reporting of all CD4 and VL test results to the state or local health department, 2) ≥95% of all laboratory test results were reported by laboratories that conduct HIV-related testing for each jurisdiction, and 3) the jurisdiction reported to CDC all CD4 and VL results received since at least January 2010.

¶ Defined as having one or more CD4 (count or percentage) or VL test performed within 3 months after HIV diagnosis during 2010, including those performed during the same month as diagnosis.

** Defined as having two or more CD4 or VL results at least 3 months apart during 2010, among persons diagnosed through December 31, 2009, and alive on December 31, 2010.

†† ART prescription was based on MMP data for all black MMP participants in the 2010 data collection cycle.

§§ Viral suppression was based on all black MMP participants in the 2010 data collection cycle and was defined as having a VL result of ≤200 copies/mL at the most recent HIV VL in the preceding 12 months. The cut-off value of ≤200 copies/mL was based on the U.S. Department of Health and Human Services recommended definition of virologic failure.

¶¶ Additional information available at http://www.cdc.gov/nchhstp/newsroom/hivfactsheets/future/high-impact-prevention.htm.

*** Additional information available at http://www.cdc.gov/hiv/prevention/demonstration/capus.
